# Analysis of cerebrospinal fluid protein concentrations of patients with cryptococcal meningitis treated with antifungal agents

**DOI:** 10.1186/s12879-015-1063-0

**Published:** 2015-08-13

**Authors:** Liang Huang, Hui Ye, Junyan Qu, Yanbin Liu, Cejun Zhong, Guangmin Tang, Ying Liu, Yao Huang, Xiaoju Lv

**Affiliations:** Center of Infectious Diseases, West China Hospital, Sichuan University, No. 37 Guo Xue Xiang, Chengdu, 610041 Sichuan China

**Keywords:** Cryptococcal meningitis, Cerebrospinal fluid protein concentration, Antifungal treatment

## Abstract

**Background:**

Many neurological diseases are accompanied by an increase in the cerebrospinal fluid (CSF) protein concentration, which indicates dysfunction of the blood-CSF/blood–brain barrier. However, the significance CSF protein concentration of patients with cryptococcal meningitis (CM) is not fully understood. The aim of the present was to determine whether CSF protein concentrations correlated with the responses of patients to treatment with antifungal drugs.

**Methods:**

We conducted a retrospective study of the analytical data of 623 lumbar punctures of 46 patients with CM who were treated at West China Hospital. We divided the patients into groups with good or poor responses to antifungal treatment. We used a generalized linear mixed model (GLMM) to evaluate the significance of the differences between the two groups.

**Results:**

The baseline CSF protein concentrations of the good antifungal response group (GR-group) (median = 0.97 g/L) were higher compared with those of the poor antifungal response group (PR-group) (median = 0.72 g/L). Analysis using the GLMM indicated that the CSF protein concentration of the GR-group decreased at a rate of 1.8 mg/L per day after antifungal treatment started and was 2.1 mg/L higher compared with that of the PR-group.

**Conclusions:**

Compared with poor responders, we found that the baseline CSF protein concentrations of good responders were higher and decreased at faster rate after the initiation of antifungal treatment.

## Background

Cryptococcal meningitis (CM) caused by *Cryptococcus neoformans* and *Cryptococcus gattii* [1] is notorious for prolonged treatment and high mortality [2–4]. There is an increasing number of cases caused by *Cryptococcus gattii*, which infects both immunosuppressed and immunocompetent individuals [5]. In contrast, there are reports of immunocompetent patients infected with *C. neoformans* [6–8]. This disease imposes a great burden worldwide, particularly in sub-Saharan Africa [3].

Lumbar puncture (LP) is advocated for the diagnosis and management of patients with CM [9, 10]. Analyses of the cerebral spinal fluid (CSF) of patients with CM patients provide a wealth of clinical data acquired through the use of the India ink stain, microbial culture, and biochemical tests. The CSF protein concentration is important as well, because increased concentrations are present in patients with neurological diseases. This condition is referred to as blood-CSF/blood–brain barrier dysfunction [11]. However, the clinical implication of differences in CSF protein concentrations in patients with CM is not fully understood.

The clinical interpretation of the levels of CSF protein concentrations is difficult, because the values change during treatment, and most studies report a single data point data rather than a time course. In the present study, we conducted a retrospective analysis of patients with CM who received antifungal treatment in our hospital and evaluated the clinical implication of the longitudinal data of CSF protein concentrations and the association with patients’ outcomes.

## Methods

### Patients and definitions

We analyzed the data for 46 patients who were admitted to West China Hospital from 2009 to 2014. CM was diagnosed according to clinical symptoms and a positive culture of the CSF or the results of the India ink stain. Patients with CM were included in this study if they met all the criteria as follows: 1) monitored for more than 30 days, 2) treated with antifungals for no longer than 7 days before admission, 3) not suspected of intracranial infection with other pathogens, and 4) CSF data acquired on a regular basis. All patients were administered antifungal treatment according to ithe guidelines of the Infectious Diseases Society of America [9]. Typically, patients require multiple LPs for regular follow-up after discharge. The Ethics Committee in West China Hospital approved this retrospective study.

### LPs and assay of CSF protein concentration

LPs were routinely performed approximately once each week for each patient. The CSF samples were immediately sent to the laboratory and analyzed for cryptococcal antigen, cell counts, and biochemical parameters. India ink staining and fungal cultures were performed as well. The India ink stain, fungal cultures, biochemical tests, and cell counts were compulsory and the other tests were optional. The CSF protein concentration data for each patient were sorted in chronologically with the baseline data (before antifungal treatment) listed first for each patient.

### Definitions of patients’ responses to antifungal drugs

We aimed to explore the association between patients’ responses to treatment with antifungal drugs and CSF protein concentrations. Patients were assigned to the poor antifungal response group (PR-group) according to the criteria as follows: persistent positive culture or positive India ink stain of the CSF 30 days after initiation of antifungal therapy. The other patients were assigned to the good antifungal response group (GR-group).

### Statistical analysis

A generalized linear mixed model (GLMM) was used for the statistical analysis. This model is suitable for analyzing repeated measures data [12]. The data acquired using the GLMM were analyzed using R software [13] and the nlme [14] and ggplot2 [15] packages. The values of categorical variables are represented as frequencies, and the values of continuous variables are presented as the mean, standard deviation (SD), range, or median values. The Student *t* test and the Wilcoxon rank sum test were used to evaluate the significance of differences between the values of continuous variables. Spearman’s rank correlation test was used to test bivariate correlations. The Fisher’s exact test were used to evaluate the significance of differences between the values of categorical variables.

## Results

There were no significant differences between age, sex, and underlying diseases of the GR and PR groups (Table 1). The observation periods ranged from 32 days to 457 days. Patients received antifungal treatment within several days after admission. Two patients died while hospitalized.

**Table 1:** Demographics and underlying diseases of patients with CM

Variables	PR-group (*n* = 28)	GR-group (*n* = 18)	*P*-value
Age, year			
Mean/SD	35.07/12.48	41.00/13.67	0.15
Range (min-max)	16-60	18-67	-
Sex, male	19(67.9 %)	13(72.2 %)	1.00
Diabetes mellitus	2(7.14 %)	2(11.1 %)	0.63
Kidney diseases	1(3.57 %)	0(0.00 %)	1.00
Autoimmune diseases	3(10.7 %)	2(11.1 %)	1.00
HIV positive	2(7.14 %)	2(11.1 %)	0.64
Malignancy	1(3.57 %)	0(0.00 %)	1.00
Hypertension	0(0.00 %)	1(5.56 %)	0.43
Respiratory infection	8(28.6 %)	4(22.2 %)	0.74
Cardiovascular diseases	1(3.57 %)	0(0.00 %)	1.00
HBV positive	4(14.3 %)	1(5.56 %)	0.64

Clinical treatment of patients with CM employs amphotericin B (AMB) and flucytosine to decrease the fungal burden in the central nervous system (CNS) [16]. To achieve a better outcome, most patients receive treatment that includes AMB [17]; however, we found that the implementation of antifungal strategies varied because of practical considerations such as costs, side effects, and availability of drugs (e.g. flucytosine was temporarily unavailable). The antifungal strategies are listed in Table 2, and analysis using the Fisher's exact test did not reveal significant differences between the groups.

**Table 2:** Antifungal treatment strategies

Antifungal strategies	PR-group (*n* = 28)	GR-group (*n* = 18)	*P*-value
AMB	1(3.57 %)	1(5.56 %)	1.00
AMB+ flucytosine	5(17.9 %)	5(27.8 %)	0.73
AMB+ fluconazole	5(17.9 %)	2(11.1 %)	0.70
AMB+ fluconazole + flucytosine	13(46.2 %)	7(38.9 %)	0.79
AMB+ voriconazole	0(0.00 %)	1(5.56 %)	0.44
Fluconazole	3(10.7 %)	0(0.00 %)	0.29
Fluconazole + flucytosine	0(0.00 %)	2(11.1 %)	0.17
Voriconazole	1(3.57 %)	0(0.00 %)	0.62

### Responses to antifungal treatment

Thirty days after initiating antifungal treatment, the CSFs of 18 (39.1 %) of 46 good responders were sterile, and the India ink stains were positive for the other 28 (60.9 %) patients. However, the clinical responses of the groups did not correspond completely with the CSF data described below.

### CSF protein concentrations

**Fig. 1:**
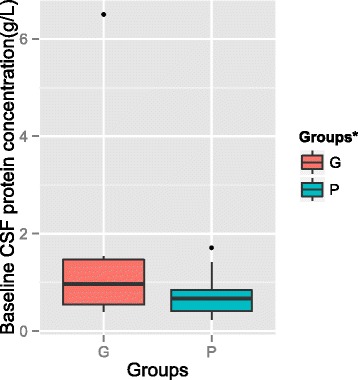
Baseline CSF protein concentrations. The difference between groups was statistically significant (*P* = 0.04). ^*^G: GR-group; P: PR-group

Analysis using the Wilcoxon rank sum test revealed significant difference between the median values of baseline CSF protein concentrations of the groups (*P* = 0.04) (Fig. 1). The median value of the CSF protein concentration of patients in the good response group was 0.97 g/L and was >0.45 g/L (the upper limit of the normal value) for 16 patients (88.8 %). In contrast, median value of the CSF protein concentrations of patients in the poor antifungal response group was 0.72 g/L, and the CSF protein concentrations were >0.45 g/L for 18 (64.2 %) patients.

**Fig. 2:**
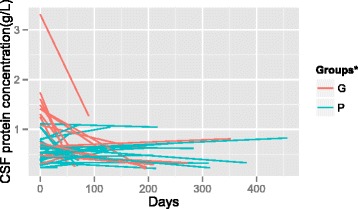
CSF protein concentrations after initiation of antifungal therapy. ^*^G: GR-group; P: PR-group

Analysis using the GLMM revealed the relationship between CSF protein concentrations and time (Fig. 2). Further, the data indicated that the CSF protein concentrations of patients in the good antifungal response group decreased at an average rate of 1.8 mg/L per day after antifungal therapy started. This value was 2.1 mg/L higher compared with that of the poor antifungal response. The CSF protein concentration of the GR-group before antifungal treatment was 0.8500 g/L, 0.3221 g/L higher compared with that of the PR-group (Table 3). These results indicate that the CSF protein concentrations were higher among the patients in the good antifungal response group before therapy started and that the concentrations decreased faster compared with those among patients in the poor antifungal response group (Fig. 2).

**Table 3:** Parameters of the GLMM estimation

Parameters	Estimation	SE	*P*-value
Intercept	0.8500	0.0755	0.0000
Time (days)	−0.0018	0.0004	0.0000
Groups^#^	−0.3221	0.0847	0.0004
Times (days) by Groups^#^	0.0021	0.0005	0.0000

^#^0 = GR-group; 1 = PR-group

## Discussion

We show here that patients in the good antifungal response group had higher baseline CSF protein concentrations, which indicates an increased inflammatory response in the CNS [18]. A plausible hypothesis to explain the data is that the intensity of the inflammatory response influences the antifungal response, and the CSF protein concentrations simply represent the intensity of inflammatory responses. Another study of patients with CM found that those infected with hepatitis B virus (HBV) had lower CSF white blood cell (WBC) counts and lower percentages of total protein in the CSF > 0.45 g/L, indicating a lower intensity of immune inflammation compared with patients with CM who were not infected with HBV who had a lower survival rate [19]. These findings indicate a correlation between the intensity of inflammatory responses and clinical outcomes.

A prospective study of patients with CM indicates that the baseline number of CSF colony-forming units (CFUs) is a prognostic factor, because patients with a lower number of CSF CFUs had better clinical outcomes [20]. Another study found a negative correlation between protein concentrations and fungal burdens in the CSF [21]. The higher baseline CSF protein concentrations reported here indicate a lower fungal burden, which indicated a better antifungal response. Others found that a higher WBC correlates with a good clinical response [21, 22] as well as a positive correlation between WBC count and CSF protein concentration was found in this study(Spearman’s rank correlation: R = 0.34, *P* = 0.00). Because drug concentrations in the CSF may be higher during inflammation of the CNS compared with those of patients without inflammation [23, 24], an elevated CSF protein concentration might indicate a higher level of inflammation that increases the transport of AMB to the CNS [24]. The rapid decrease of the CSF protein concentration in the GR-group indicates faster attenuation of inflammation, which led to a more effective antifungal response.

There are differences between the findings of the present study and those of other reports. For example, Lu et al., (1999) reported no significant difference in CSF protein concentrations between treatment failures and cured or improved groups [22]. However, the value of a single LB was not sufficient to evaluate the significance of changes in CSF protein concentrations during treatment. The difference may be attributed to the type of data analysis used compared with that of the present study. In the present study, we determined the time course of changes in CSF protein concentrations. Patients with CM are frequently immunocompromised, for example, those with infected with HIV [1]. However, only four (8.70 %) patients studied here were infected with HIV, which is lower compared with the rates reported by others [25]. The multiple polymorphisms in the genes encoding mannose-binding lectin and the Fc-gamma receptor 2B (FCGR2B) in the Han population, which is the largest ethnic group in China, might contribute to the discrepancies between studies [26]. The mortality rates (4.34 %) of patients treated at our hospital are lower compared with those of other studies [1]. The lower proportion of HIV-related infections may explain the discrepancy as well, and observations over eight years reveal that mortality rates are lower for patients with CM who are not infected with HIV [27].

## Conclusions

The present study supports the hypothesis that there is a correlation between antifungal responses and CSF protein concentrations. These findings must be confirmed by prospective studies of larger numbers of patients. However, we recommend that the treatment of patients with CM should include detailed analyses of CSF protein concentrations with focus on their variability.

## Abbreviations

CFUs: Colony-forming units
SD: Standard deviation
SE: Standard error
WBC: White blood cell
HIV: Human immunodeficiency virus
